# A data set of distributed global population and water withdrawal from 1960 to 2020

**DOI:** 10.1038/s41597-022-01760-1

**Published:** 2022-10-21

**Authors:** Denghua Yan, Xin Zhang, Tianling Qin, Chenhao Li, Jianyun Zhang, Hao Wang, Baisha Weng, Kun Wang, Shanshan Liu, Xiangnan Li, Yuheng Yang, Weizhi Li, Zhenyu Lv, Jianwei Wang, Meng Li, Shan He, Fang Liu, Wuxia Bi, Ting Xu, Xiaoqing Shi, Zihao Man, Congwu Sun, Meiyu Liu, Mengke Wang, Yinghou Huang, Haoyu Long, Yongzhen Niu, Batsuren Dorjsuren, Mohammed Gedefaw, Yizhe Li, Zihao Tian, Shizhou Mu, Wenyu Wang, Xiaoxiang Zhou

**Affiliations:** 1 State Key Laboratory of Simulation and Regulation of Water Cycle in River Basin, China Institute of Water Resources and Hydropower Research, No. 1 Fuxing Road, Haidian District, Beijing, 100038 China; 2 State Key Laboratory of Hydrology-Water Resources and Hydraulic Engineering, Nanjing Hydraulic Research Institute, Nanjing, 210029 China

**Keywords:** Water resources, Hydrology

## Abstract

Population and water withdrawal data sets are currently faced with difficulties in collecting, processing and verifying multi-source time series, and the spatial distribution characteristics of long series are also relatively lacking. Time series is the basic guarantee for the accuracy of data sets, and the production of long series spatial distribution is a realistic requirement to expand the application scope of data sets. Through the time-consuming and laborious basic processing work, this research focuses on the population and water intake time series, and interpolates and extends them to specific land uses to ensure the accuracy of the time series and the demand of spatially distributed data sets. This research provides a set of population density and water intensity products from 1960 to 2020 distributed to the administrative units or the corresponding regions. The data set fills the gaps in the multi-year data set for the accuracy of population density and the intensity of water withdrawal.

## Abstract (summary)

**Table:** 

Measurement(s)	distributed global population and water withdrawal
Technology Type(s)	mathematical statistics and analysis

## Background & Summary

The rapid increase in global water use can be attributed to factors such as population growth and economic development^1^. the world’s population still continues to grow, albeit at a slowing rate, but there are significant differences in regional characteristics^2^. The increase in population and water withdrawal demand makes it difficult to make decisions about water allocation, and the demand competition between population and water withdrawal further exacerbates the risk of regional conflicts^3^. Identifying, measuring and expressing the value of water is the focus of the United Nations World Water Development at this stage^2^, but it is also inseparable from the research on the changes in world population development and water withdrawal patterns, especially the demand for relevant long basic data sets. Obtaining sector and high-precision reference data through deep mining has taken a key step in the refinement of water withdrawal^4–6^, but long series of basic data usually come from FAO and other international organizations. Their data is often time-periodized and discrete, and there is almost no continuous long series of population and water withdrawal data sets globally^7^.

It is also an indisputable fact that the data published by international organizations do have errors (There is data duplication in different time periods^8^). The population or water withdrawal data released by the World Bank, FAO and other international organizations may deviate from the real population or water withdrawal data of a country or region due to statistical caliber, and its time series accuracy needs to be further corrected^9–11^. We believe that the statistical data of relevant departments in various countries are more authoritative, but the collection and other work are very difficult. This data set collects, interpolates and extrapolates the data published by government agencies to achieve horizontal expansion of the basic data. It is the foundation of the follow-up research work and the work that cannot be ignored to fundamentally improve the accuracy of basic data.

Basic work of collection, induction and interpolation are carried out for individual countries, and it is difficult to establish spatial connections. Considering the need for spatialization of basic data and comparability of regional development, two variables of population density and water intensity^11^ were introduced. Among them, population density refers to the population per unit area (person /km^2^), and water intensity refers to the water withdrawal per unit area (m^3^/km^2^). Refined spatial classification is still a difficult problem at this stage. This data set only realizes spatial distribution on administrative units and land use. If there is a more refined classification, spatial connections can be established independently based on the population and water withdrawal data sets in this data set.

**Fig. 1:**
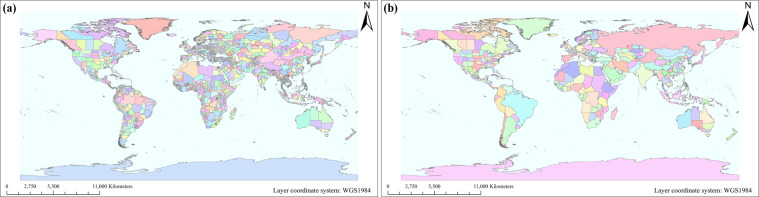
The spatial boundaries of population density (**a**) and water intensity (**b**).

This data set includes a set of population density products distributed to the administrative units from 1960 to 2020, a set of water intensity products distributed to the administrative units, a set of population density products distributed to an artificial surface, a set of water intensity products distributed to the artificial surface and cultivated land, an EXCEL file for the revised population of different countries, and an EXCEL file for the total amount of water withdrawal of different countries (Tables 1 and 2). Considering that the naming of low-level administrative units in different countries is different, the concept of sub-national is adopted in this set data, and the spatial boundaries of population density and water intensity are shown in Fig. 1.

**Table 1:** Input data sets used to produce the global population and water withdrawal products.

Name	Acquisition Year	Source	Spatial Resolution	Format	Spatial Reference	URL
Globeland 30	2000/2010	National Geomatics Center of China (NGCC)	1” (~30 m)	/	GCS WGS 1984	https://www.webmap.cn/main.do?method=index
National boundaries	2015	National Geomatics Center of China (NGCC)	1 km	ESRI polygon shapefile	GCS WGS 1984	
Subnational boundaries	2015	National Geomatics Center of China (NGCC)	1 km	ESRI polygon shapefile	GCS WGS 1984	
Population	1960~2020	World Bank	/	EXCEL	/	https://data.worldbank.org/indicator/SP.POP.TOTL
	1960~2020	Food and Agricultural Organization	/	EXCEL	/	https://www.fao.org/aquastat/statistics/query/index.html
Water Withdrawal	1960~2020	World Bank	/	EXCEL	/	https://data.worldbank.org/indicator/ER.H2O.FWTL.K3
	1960~2020	Food and Agricultural Organization	/	EXCEL	/	https://www.fao.org/aquastat/statistics/query/index.html
Fresh surface water abstracted	1990~2016	UN data	/	EXCEL	/	https://data.un.org/Data.aspx?q=water+&d=ENV&f=variableID%3a24
Population/ Water Withdrawal	/	Government or statistical bureau	/	EXCEL	/	10.6084/m9.figshare.19387406.v2

**Table 2:** The global population and water withdrawal products.

Name	Temporal extent	Spatial Resolution	Pixel Type & Depth	Spatial Coverage
*_dpyear_1km	1960~2020	1000 m	TIFF/flt32	Continent
*_dpyear_sr	1960~2020	1000 m	TIFF/flt32	Continent
Country_P_Data	1960~2020	/	EXCEL	Continent
*_widyear_1km	1960~2020	1000 m	TIFF/flt32	Continent
*_winyear_sr	1960~2020	1000 m	TIFF/flt32	Continent
Country_W_Data	1960~2020	/	EXCEL	Continent

Note: The relevant definitions of the terms in the table can be found in Data Records.

Data cover almost all regions of the world. Population data include 214 national units, 1805 national or sub-national units. Water data include 214 national units and 616 national or sub-national units. Because of the difficulties in obtaining regional data in some countries, sub-national data are replaced by national data. There may be some errors in the statistics and collection of the population and water withdrawal data in various countries, which may lead to deviations between the data set and the real data. Therefore, further considering the data trend of international organizations and referring to the officially released data, the accuracy of the data set of this study is sufficient to be effectively guaranteed. It can be used as the basic information for the study of global climate change, environmental resources, regional economy and political decision-making. With the improvement of the collection of relevant credible data or the accuracy of the original data acquisition in the future, the data set can be amended and supplemented.

## Methods

**Fig. 2:**
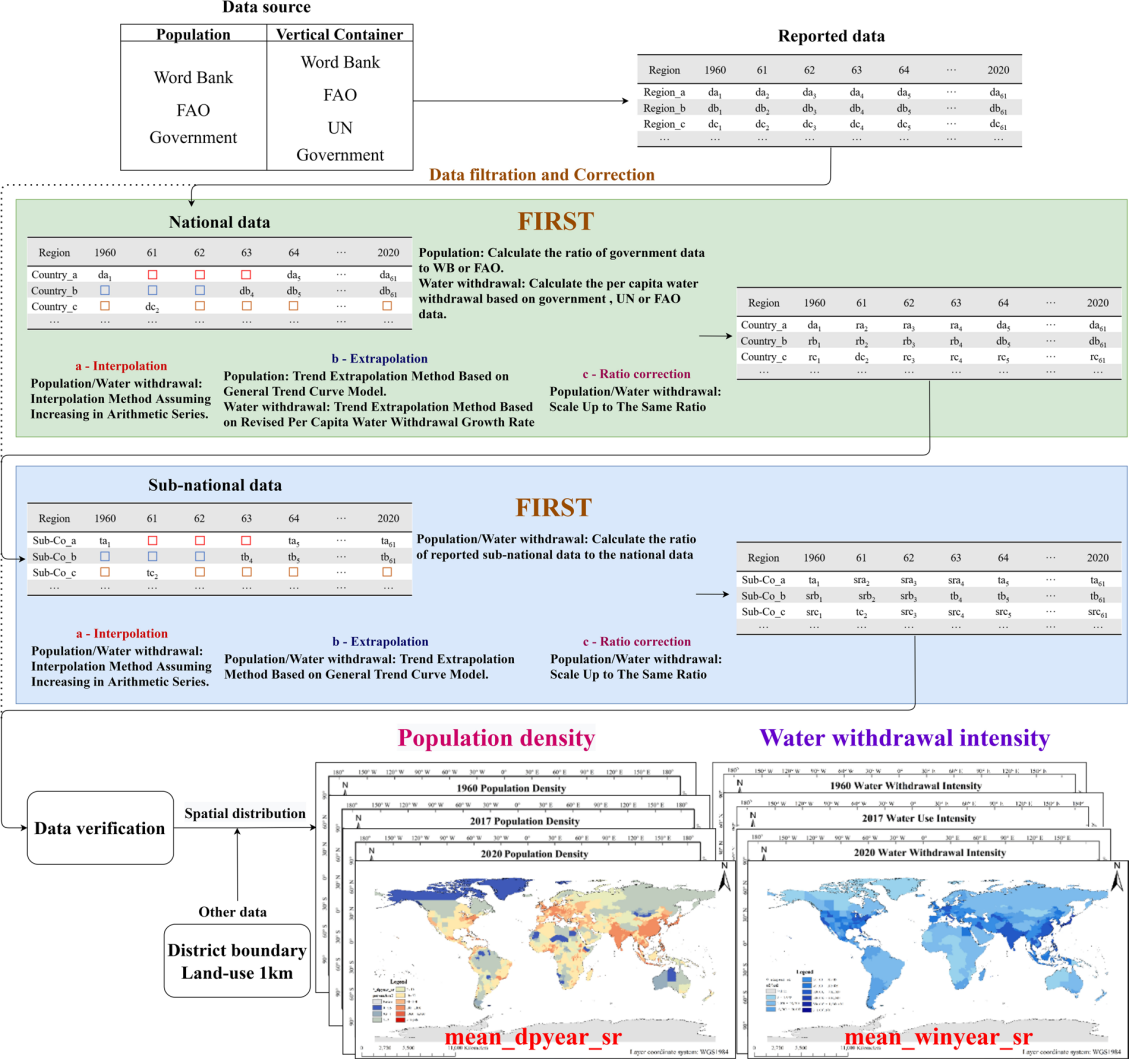
Schematic outline to produce the global population and water withdrawal products.

In this chapter, we describe in detail the method of data set generation, including data collection, data modification and interpolation extension, and grid data generation (Fig. 2).

First, the collection of population and water withdrawal data. Collect as much as possible of the national and sub-national permanent population and water withdrawal data released by governments and institutions on a global scale. Here we provide the source of our data collection.

Second, establish a national and sub-national default data interpolation model. Based on the shape of the sample data scatter plot, determine the most appropriate curve model. The simulation modeling is implemented by EXCEL and provided one by one according to the national level.

Third, create spatial distribution grids. Spread the population density to the administrative unit and artificial surface, and spread the water intensity to the administrative unit, and artificial surface and cultivated land (Spatial distribution section for details).

Fourth, data verification. For population data, we compare the global population of the revised results with data of the World Bank and FAO, and calculate the correlation and deviation between the revised results and the other two sets of data. For the water withdrawal data, we divide the measured data into calibration and verification periods, re-interpolate the data using the data of the calibration period, and then verify the simulation accuracy by using the data of the verification period and the simulation.

### Data collection and pretreatment

The data sources include government population data for xx nation and xx sub-nation, government water withdrawal data for xx nations and xx sub-nations, national population and water withdrawal data from the World Bank^12^, and national population and water withdrawal data from FAO^8^, water withdrawal data from the United Nation^13^, national population and water withdrawal data from Eurostat^14^, and Globeland30^15^ data for 2000 and 2010. Among them, xx refers to one of many countries in the data set, and only serves as an indicator.

Globally, it is believed that the accuracy rate of census results obtained by counting the population of various administrative units in the country is the highest at present when a large amount of manpower and material resources are spent by the country itself^16^. In addition to the census conducted every certain year, the statistical department gets a high accuracy rate by calculating the overall figures according to the sample survey of population changes and the random sample survey of fertility rate in some areas and some units. To sum up, we believe that the data released by our country on the statistical official website is the most reliable.

When national population data are missing, it is generally believed that the data and trends of the World Bank and FAO are authoritative. When the data of the World Bank and FAO are complete, the World Bank data prevails as reference population data. When the length of World Bank data is shorter that of than FAO, the FAO data is used as reference population data^17^.

For water withdrawal data, FAO and UN data are generally considered authoritative when government water withdrawal data is missing. When the FAO and UN data are both complete, the FAO data is used as a reference for water withdrawal data.

### Interpolation and extrapolation of national and sub-national population data

When the lack of data is obvious, the results obtained by the simplest method often have more reference value. The following four basic methods are used for the processing of population data^9–11,18–20^.

#### Interpolation method assuming increasing in arithmetic series

If discontinuities exist in government data, and the number of data increases in arithmetic series according to the judgement, then the linear interpolation method can be used based on a linear model of arithmetic series growth. This method is suitable for interval data interpolation with a short interruption time and relatively uniform data growth scale. The interpolation model is as follows:

1 $${P}_{N,k}=\left[\frac{I\left(j\right)-I\left(i\right)}{j-i}\cdot \left(k-i\right)+I\left(i\right)\right]\cdot {P}_{W,k}$$

Where, *P*_*N,k*_ is the government data for the *k* year, *i* ≤ *k* ≤ *j*; *P*_*W,k*_ is the reference data for the *k* year; *I*(*j*) and *I*(*i*) are the ratios of government data to reference data for the *j* year and *i* year, respectively.

#### Trend extrapolation method based on general trend curve model

If there are continuous points in the government data, it is better to obtain interpolation results by assisting based on the trend of the ratio of government data to reference data. General trend line functions such as linear, conic, cubic and exponential curves can be used, and the fitting result needs to be comprehensively judged by the linear change of the reference data, and finally a more suitable interpolation result can be obtained. This method is more suitable for interval data interpolation with shorter time and faster data growth.

2 $${P}_{N,k}=F(k)\cdot {P}_{W,k}$$

where, *P*_*N,k*_ is the government data in the *k* year, *i* ≤ *k* ≤ *j*; *P*_*W,k*_ is the reference data for the *k* year; *F*(*k*) is the trend for the ratio of government data to reference data in the *k* year.

#### Scale up to the same ratio

If there is only one year of government data, then the reference data will be scaled up to the same ratio according to the ratio of government data to the reference data of the corresponding year.

3 $$I=\frac{{P}_{N}}{{P}_{W}},{P}_{N,o}=I\cdot {P}_{W,o}$$

Where, *P*_*N*_ is the government data; *P*_*W*_ is the reference data; *I* is the ratio of government data to reference data; *P*_*N,o*_ is the default government data; *P*_*W,o*_ is the reference data corresponding to the default; *o* is the default year.

#### Based entirely on government data or reference data

If there is complete government data, the government data is used as the final population result. If there is no government data, the reference data is used as the final result of the population.

### Interpolation and extrapolation of national and sub-national water withdrawal data

The total amount of water withdrawal in various countries varies greatly, but the per capita water withdrawal of the country generally remains within a certain range. Therefore, we first calculate the reference data, and then interpolate and extrapolate the missing per capita water withdrawal data. The methods can also be summarized into the following five categories.

#### Interpolation method assuming increasing in arithmetic series

The calculation principle is the same as the interpolation method of national population data. This method is more suitable for interval data interpolation with shorter and discrete data, such as the data form before 1990 in Fig. 6(c).

#### Trend extrapolation method based on revised per capita water withdrawal growth rate

If there are continuous points in the data, we assume that the per capita water withdrawal versus time curve is consistent with the S curve, that is, the per capita water withdrawal shows only a slow change in the first years and the last years. We first calculate the growth rate of per capita water withdrawal in the last two years or the first two years, adjust the final growth rate proportionally to reflect the subsequent changes, and adjust the first growth rate proportionally to reflect the previous changes. Equation (4) represents a method of extrapolating the previous missing value data, and Eq. (5) represents a method of extrapolating the subsequent missing value data. This method is more suitable for the situation where continuous government data exists and the change trend of per capita water consumption is clear, such as the form of continuous data after 1990 in Fig. 6(c).

4 $$\left\{\begin{array}{rll}{s}_{i} & = & \frac{{w}_{i}-{w}_{i+1}}{{w}_{i+1}}\\ {s}_{i-1} & = & {s}_{i}\cdot (1-\theta )\\ {w}_{i-1} & = & {w}_{i}\cdot (1+{s}_{i-1})\end{array}\right.$$

5 $$\left\{\begin{array}{rll}{s}_{j} & = & \frac{{w}_{j}-{w}_{j-1}}{{w}_{j-1}}\\ {s}_{j+1} & = & {s}_{j}\cdot \left(1-\theta \right)\\ {w}_{j+1} & = & {w}_{j}\cdot \left(1+{s}_{j+1}\right)\end{array}\right.$$

Where *w*_*i-*1_ is the missing per capita water withdrawal value for time step *i-*1; *s*_i-1_ is the missing reverse order growth rate value for time step *i*-1; *w*_*i*_ and *w*_*i+*1_ are the first two known per capita water withdrawal values for time step *i* and *i* + 1, and *s*_i-1_ is the known reverse order growth rate value for time step *i*-1. For Eq. (5), *w*_*j+*1_ is the missing per capita water withdrawal value for time step *j* + 1; *s*_*j+*1_ is the missing growth rate value for time step *j* + 1; *w*_*j-*1_ and *w*_*j*_ are the last two known per capita water withdrawal values for time step *j* and *j*-1, and *s*_*j*_ is the known growth rate value for time step *j*. To ensure that the per capita water withdrawal in the front of the series or in the latter part of the series does not change too fast, the equation introduces *θ* to represent the correction coefficient for the growth rate, which is generally in the range of 0.1 to 0.2.

#### Scale up to the same ratio or smoothing spline fitting

If there is only one data released, the per capita water withdrawal of that year will be used for all years. For water withdrawal data with long time spans and more data but many intervals, we use smoothing spline to provide smooth interpolation over time, taking into account the equilibrium of per capita water withdrawal fluctuations.

#### Proximity of adjacent region

If no national water withdrawal data is released, based on the country’s level of development and geographic location, the per capita water withdrawal of adjacent countries with similar development levels is selected as an approximate value for the country’s per capita water withdrawal value.

The treatment of sub-national water withdrawal data is similar to sub-national population data. First, the ratio of the sub-national data to the national data of the known year is calculated, and then the interpolation and extrapolation methods are used to calculate the ratio of the missing values, and finally sub-national data is obtained by the national data and the ratio.

### Spatial distribution

**Fig. 3:**
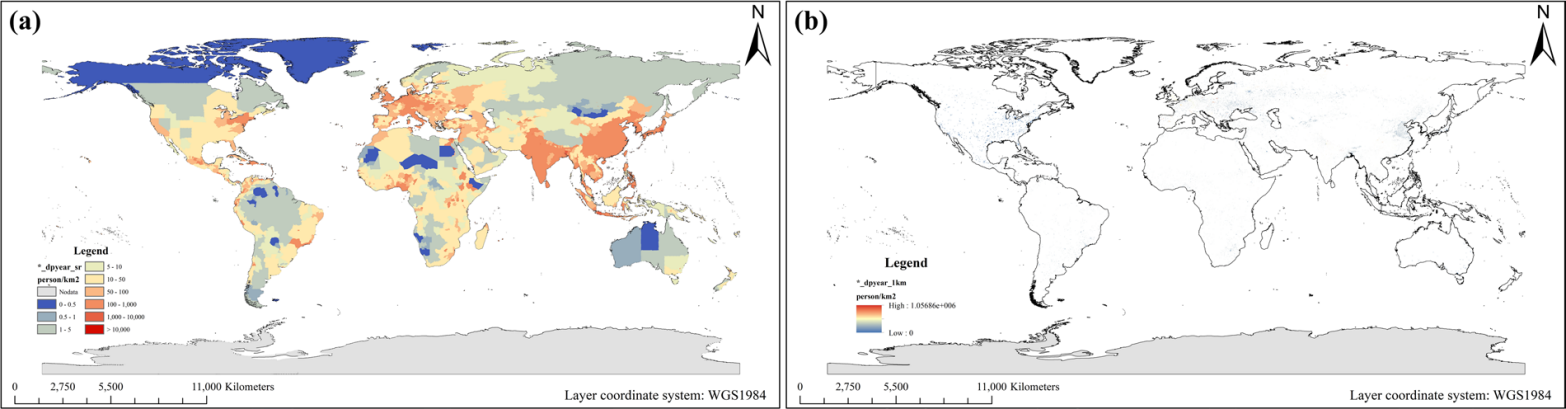
The specific regional average population density from 1960 to 2020. (**a**) The administrative units. (**b**) The artificial surface grids. Obtain the population of the above-mentioned two groups of specific regions within each 1 km grid in an average manner.

**Fig. 4:**
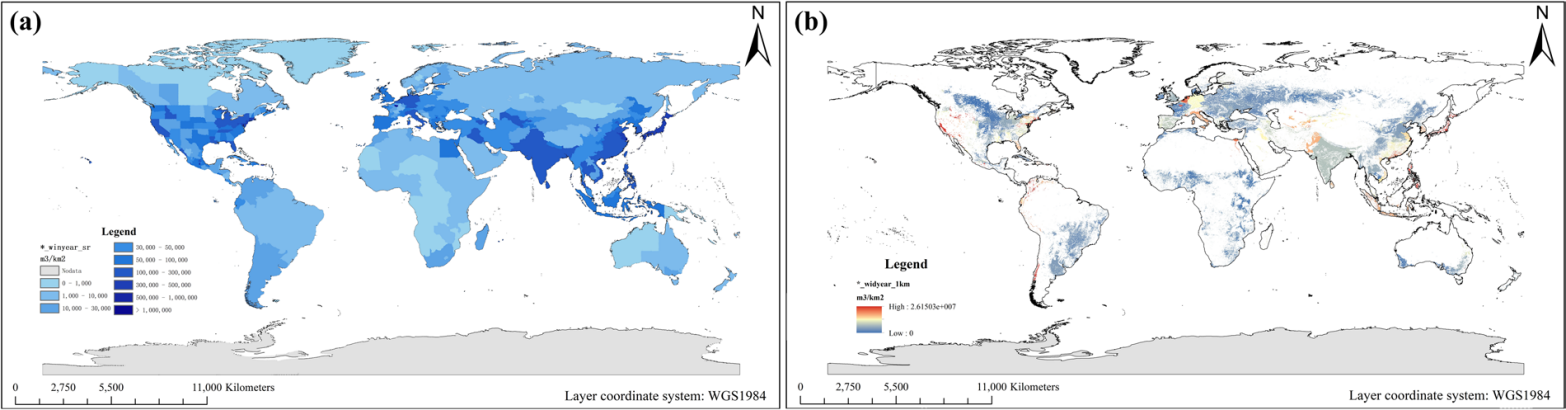
The specific regional average water intensity from 1960 to 2020. (**a**) The administrative units. (**b**) The artificial surface and cultivated land grids. Obtain the water withdrawal of the above-mentioned two groups of specific regions within each 1 km grid in an average manner.

This research further considers the indicative role of specific land use types. Spatial distribution, which means that the data is distributed to a meaningful area. It is assumed that the population and water are only used on an artificial surface and cultivated land. We mainly used the globeland30 data^15^ of 2000 and 2010 to process the data before and after 2000, respectively (Figs. 3 and 4).

Based on ArcGIS Desktop 10.2, convert the global land use grid into a vector format, and then extract the global artificial surface and cultivated land. The population density and water intensity on the grid are expressed as follows^21^:

6 $$S{D}_{ad,P}=\frac{{P}_{ad}}{{A}_{ad}},S{D}_{lu,P}=\frac{{P}_{ad}}{{A}_{lu,a}}$$

7 $$S{D}_{ad,W}=\frac{{W}_{ad}}{{A}_{ad}},{SD}_{lu{\rm{,}}W}=\frac{{W}_{ad}}{{A}_{lu,ac}}$$

Where, *SD*_*ad, P*_ and *SD*_*ad*, *W*_ are the population density and water intensity of an administrative unit, respectively; *SD*_*lu, P*_ is the population density on the artificial surface of an administrative unit; *SD*_*lu, W*_ is the water intensity on the artificial surface and cultivated land of an administrative unit; *P*_*ad*_ and *W*_*ad*_ are the population and water withdrawal of an administrative unit, respectively; *A*_*ad*_, *A*_*lu, a*_ and *A*_*lu, ac*_ are the area of an administrative unit, the area of the artificial surface of an administrative unit, and the area of artificial surface and cultivated land of an administrative unit.

## Data Records

The output data sets described in this article are publicly and freely available through the website^22^. The data set includes 4 sets of raster data and 2 sets of EXCEL spreadsheet data, which are published separately on each continent. Abbreviations for each continent are as follows: NA-North America, SA-South America, EU-Europe, AS-Asia, AF-Africa, OC-Oceania. The data includes the following:

*_dpyear_sr: Spatial distribution of population density grid data sets in national or sub-national administrative units, with a unit of person/km^2^.

*_dpyear_1 km: Spatial distribution of population density grid data sets on 1 km resolution artificial surface grids, with a unit of person/km^2^.

*_winyear_sr: Spatial distribution of water intensity grid data sets in national or sub-national administrative units, with a unit of m^3^/km^2^.

*_widyear_1km: Spatial distribution of water intensity grid data sets on 1km resolution artificial surface and cultivated land grids, with a unit of m^3^/km^2^.

Country_P_Data.xlsx: processing and result documents of national population data, including year, World Bank data, FAO data, the government published data, and revised population data, with a unit of 10,000 people.

Country_W_Data.xlsx: processing and result documents of national water withdrawal data, including year, World Bank data, FAO data, UN data, the government published data, and revised water withdrawal data, with a unit of 10,000 m3.

* indicates AF, AS, EU, NA, OC and SA.

The product set is designed to fill the blanks in the long series of population and water withdrawal, enhance the accuracy of data, and can reflect the spatial distribution changes of population and water withdrawal. The data products reveal the development of the world population and the changes in the pattern of water withdrawal. They can help to reveal the regional characteristics of population development all over the world. In particular, it is of great significance to master the scale of population water withdrawal in regions where data are difficult to access.

The product offers the population density products of the minimum administrative units and artificial surface from 1960 to 2020, the products of the minimum administrative units and the artificial surface-cultivated land products, a set of EXCEL files of the revised total population in different countries, and a set of EXCEL files of the revised total water consumption in different countries.

The trend interpolation and extrapolation in the product are conducted under the assumption that the population maintains a certain natural growth and they cannot timely reflect the sudden changes in population and water withdrawal caused by major disasters (i.e. extreme floods and earthquakes), wars and large-scale migration. Given the mobility and flexibility of human activities, there may be some errors in the above data in many countries/regions. The data can be edited to meet the needs of various users. Therefore, users are encouraged to make up for the error by using recently updated data or data from specific sources.

At present, we have not collected related data of a few countries/regions, such as Mauritania, Madeira Island, St. Helena, Christmas Island, British Indian Ocean territory, the Vatican, Svalbard Island and Jan Mayen Island, Guadeloupe, St Pierre et Miquelon, Na Varsa Island, Anguilla, Montserrat, Martinique, Clipperton Island, Midway Islands, Virgin Islands, Netherlands Antilles, United States Miscellaneous Islands, Pitcairn Islands, Norfolk Island, Heard-und McDonald- Island, Bouvet Island, South Georgia and South Sandwich Island, Cocos (Keeling) Islands, Prince Edward Island, Wake Island, French Territory in The South, Falkland Islands, etc. Most of these areas are uninhabited or sparsely populated, so there are few records of water withdrawal and population data. In the data set, they are treated as no-value areas. We intend to add more data sets to the product in the future to further improve its spatial and temporal coverage.

## Technical Validation

To make the data more transparent, we have compiled detailed data sources for population and water withdrawal for each country, and the data is available as a separate EXCEL spreadsheet. These data sources, including the World Bank, FAO, the United Nations, and officially released data, are relatively accurate. The officially released data of each country is more rigorous in its own region, and we believe that the government officially publishes the highest level of accuracy. Since some of the data set was obtained by interpolation and extrapolation, we performed verification for the reliability of the data set, and the data validation graphs are provided synchronously with the data collected by each country.

**Fig. 5:**
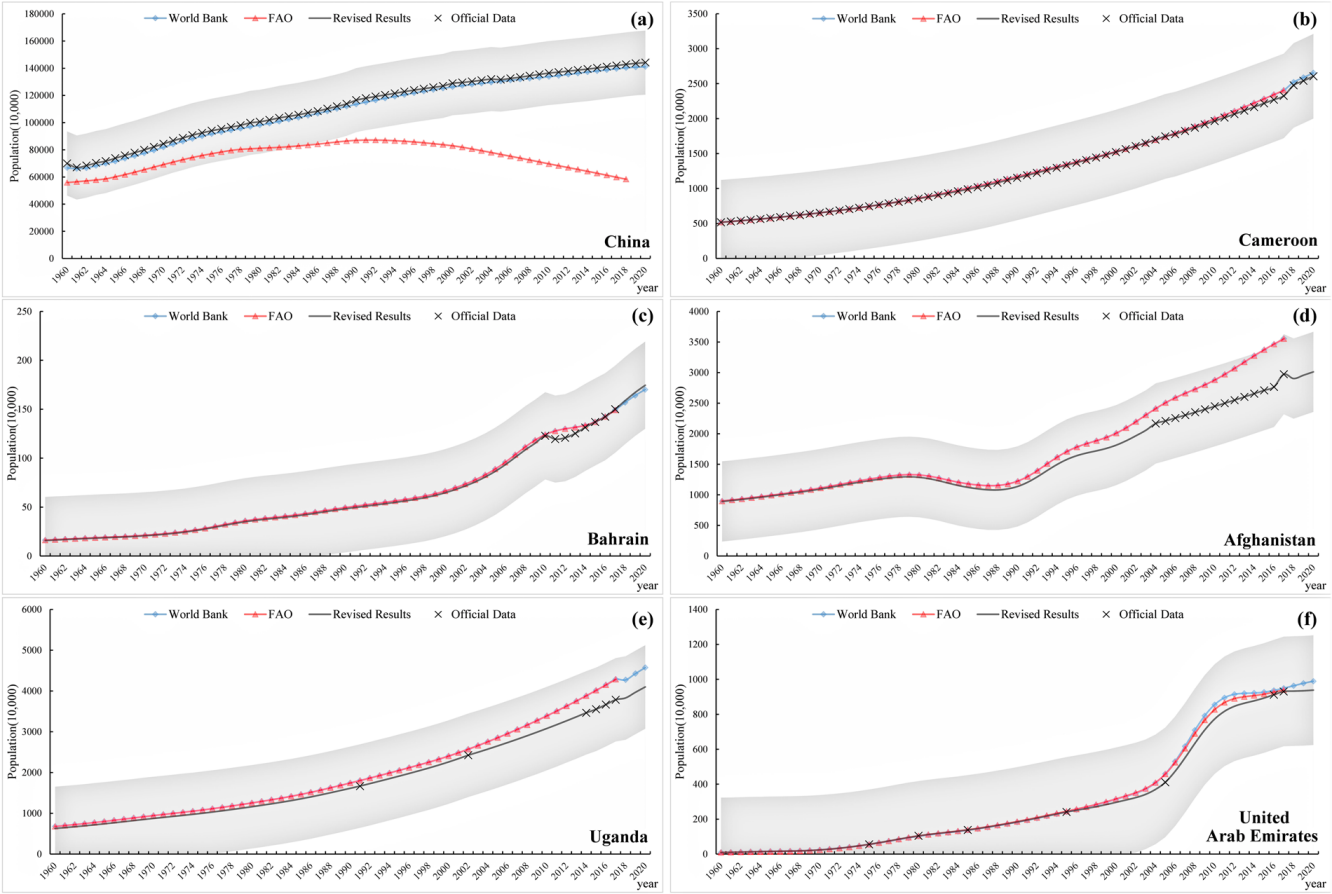
Verification of the population data. (**a**) and (**b**) represent the situation when the officially released data is relatively long. (**c**) and (**d**) represent the situation when the officially released data is concentrated. (**e**) and (**f**) represent the situation when the officially released data is discontinuous.

In the process of population data correction, the following three situations often occur (Fig. 5). When the officially released data is relatively long, we take the officially released data as the standard and revise some contents in combination with the data of international organizations; When the data series are relatively concentrated, we can only use the data and trend changes of relevant international organizations for reference to reasonably correct the missing years and make it smoothly connect the official data; When the officially released data is discontinuous, we take the official data as the correction node and learn from the trends of relevant international organizations to correct it into smooth and continuous population data.

**Fig. 6:**
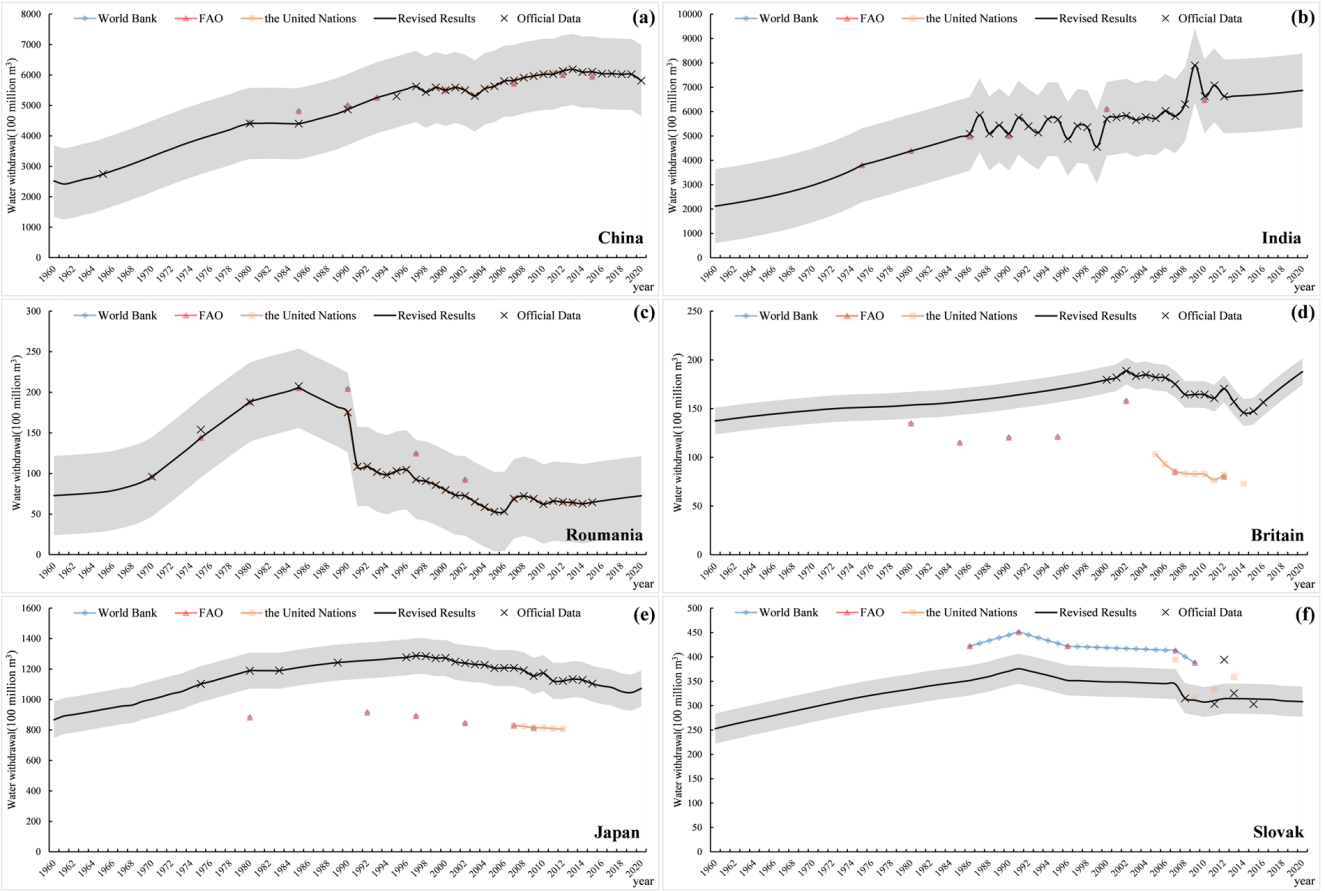
Verification of the water withdrawal. (**a**–**c**) represent cases where the official data is used as a reference because of small data differences. (**d**–**f**) represent cases where the trend changes of international organizations are used as a reference because of large data differences.

**Fig. 7:**
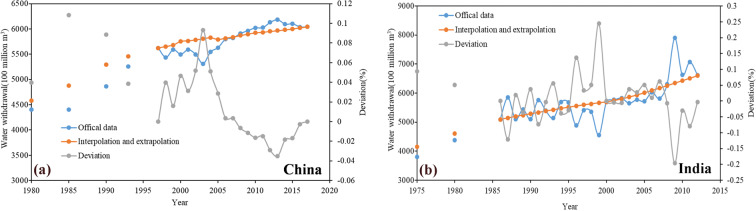
Hypothesis and verification. Select some continuous data as the calibration data, and compare the result of interpolation and extrapolation of other available data (orange) with the official calibration data (blue) to obtain the degree of deviation (gray). (**a**) represent China, the data after 1995 is selected as the verification data; (**b**) represent India, the data after 1985 is selected as the verification data.

In the process of water withdrawal data correction, we also take the officially released data as the standard. Considering the basic assumption that the water withdrawal per capita in the country or region is constant or follows a certain trend, the water withdrawal data is interpolated and extrapolated (Fig. 6). China and India have a large proportion of water withdrawal in the world and more official data, some measured data are selected for interpolation and extrapolation, and compared with the actual data (Fig. 7). The results show that the deviation of the data is mostly within ± 10%, and the reason for the large deviation of some points is due to the large annual fluctuation of official data. Therefore, it can be considered that the data set derived from the existing water withdrawal data is accurate.

## Data Availability

The data set processing process and usage method can be obtained from Figshare^22^. We believe that in the case of serious data missing or large data differences, the effect of using the most basic mathematical method is more effective and reliable. Our basic methods can be realized by using the conventional functions in Excel without new code. Please refer to the methods section. Spatial processing content ran in ArcGIS Desktop (V10.2 or later). The interpolation and extrapolation is processed in Microsoft Excel. All software needs to be installed in the Windows 10.
